# Multiscale depth of anaesthesia prediction for surgery using frontal cortex electroencephalography

**DOI:** 10.1049/htl2.12025

**Published:** 2022-05-03

**Authors:** Ejay Nsugbe, Stephanie Connelly

**Affiliations:** ^1^ Nsugbe Research Labs Swindon UK; ^2^ Hereford County Hospital Wye Valley NHS Trust Hereford UK

## Abstract

Hypnotic and sedative anaesthetic agents are employed during multiple medical interventions to prevent patient awareness. Careful titration of agent dosing is required to avoid negative side effects; the accuracy thereof may be improved by Depth of Anaesthesia Monitoring. This work investigates the potential of a patient specific depth monitoring prediction using electroencephalography recorded neural oscillation from the frontal lobe of 10 patients during sedation, where a comparison of the prediction accuracy was made across five different approaches to post‐processing; Noise Assisted‐Empirical Mode Decomposition, the Raw Signal, Linear Series Decomposition Learner, Deep Wavelet Scattering and Deep Learning features. These methods towards anaesthesia depth prediction were investigated using the Bispectral Index as ground truth, where it was seen that the Raw Signal, enhanced feature set and a low complexity classification model (Linear Discriminant Analysis) provided the best classification accuracy, in the region of 85.65 % ±10.23 % across the 10 subjects. Subsequent work in this area would now build on these results and validate the best performing methods on a wider cohort of patients, investigate means of continuous DoA estimation using regressions, and also feature optimisation exercises in order to further streamline and reduce the computation complexity of the designed model.

## 1 INTRODUCTION

Anaesthesia typically refers to the administration of a neurotropic substance with the capability of safely shifting the state of consciousness of a human being. The key goals of anaesthetic agents (also known as anaesthetics) serve as a form of muscle relaxant, to null out sensations and reflexes (analgesia), and also hypnosis, which not only involves a loss of consciousness but also a temporary loss of memory [1, 2, 3]. The roots of anaesthesia and its application in surgical interventions trace back to the BC timeline, where the earlier forms of anaesthetics ranged from herbal compounds all the way towards targeted doses of alcohol [1, 2, 3]. The first recognised and comprehensive clinical standard reference manual for anaesthesia is said to have been published in 1914 by Dr. James Tayloe Gwathmey and Dr. Charles Baskerville [4].

**FIGURE 1:**
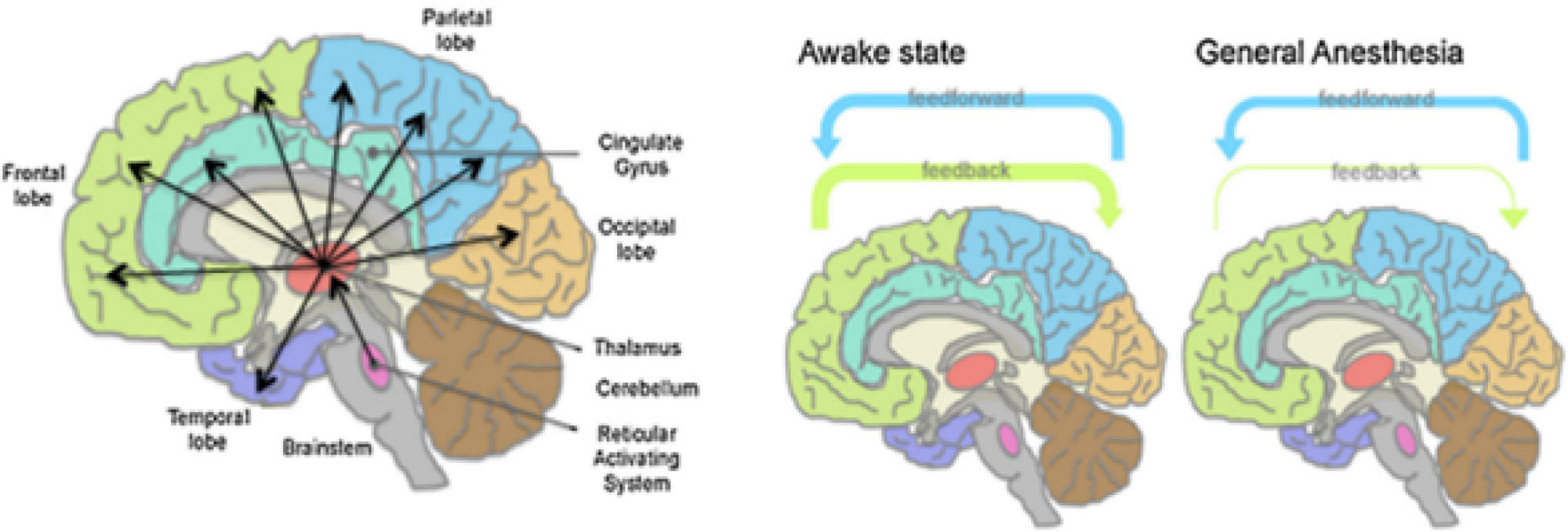
(Left) Anatomy of the brain and projections of both the thalamus and reticular system. (Right) An illustration of the reduction of sensory feedback capabilities within the brain during general anaesthetics [6]

General anaesthesia, resulting in a loss of consciousness and awareness, is required for certain types of surgery and refers to clinical substances which are capable of methodically holding various degrees of consciousness [5, 6]. There is yet to be a complete theory on the full workings of general anaesthetic agents, but the emergence of functional imaging has proven to be a useful mechanism towards studying the effects of the brain under various depths of anaesthesia [6]. Schools of thoughts around the workings of anaesthetic agents include the presence of a neural consciousness switch in the thalamus, but this has been refuted due to the consciousness deactivation pattern varying between different anaesthetic compounds [7]. An alternate candidate description on the neural workings of anaesthesia is founded on the principles of control theory, and this notion is based around the systematic nulling of the feedback path, information exchange and transfer entropy between the anterior and posterior cortical regions within the brain, as illustrated in Figure 1 [6, 8, 9].

On this basis, research has shown that the overall feedback connectivity from the anterior to posterior is strongly attenuated under the effects of general anaesthesia and subsequently begins to increase as soon as the patient begins to respond to verbal instructions, which is deemed as a marker of return to consciousness [6]. Attempts to explain the core underpinning behind the suppression of the cortical feedback during anaesthetics are based around the theory of the nulling of synaptic activities; this results in a loss of signalling which inhibits the ability for complex information processing to take place [6].

General anaesthesia is adopted as part of clinical operations, as mentioned, although there continues to be reported postoperative side effects from the use of anaesthesia which span mental anguish and instabilities alongside varying levels of cognitive dysfunctions [10, 11]. This makes it paramount for an optimal titration of the anaesthetic compound, alongside an effective means of evaluating depth of dosage in real‐time, to be of key importance in order to ensure patients’ safety [12]. The area of focus within this paper is themed around a means towards determining the optimal anaesthetic dosage based on accurate measurement of the brain's state of sedation; however, the discussion of the development of an ideal anaesthetic agent is outside the scope of this paper. The ability to accurately predict anaesthetic depth hinges on an effective ability to closely monitor the neural oscillatory states within the brain, and work is ongoing in this area [12].

Frequently adopted means which anaesthetists use towards evaluating depth of anaesthesia in patients include a combined observation of physiological parameters, that is, heart rate and blood pressure, and also a qualitative evaluation of chosen anaesthetic agents’ pharmacokinetic and pharmacodynamic effects on each patient, alongside the monitoring of the concentration of inhalation agents and/or pharmacokinetic models predicting plasma or effect site concentrations for intravenous anaesthetics [12]. Succinctly put, it can be assumed that anaesthetists rely on a fusion of autonomic and behavioural response information as a form of inference, and an estimation of brain consciousness state, which informs their strategy towards optimizing the dosage of the anaesthetic for the patient during a surgical process [12].

There has been evidence to support the notion that bioelectrical potentials from the frontal cortex, acquirable in the form of EEG, are correlated with depth of general anaesthesia [13]. As the atypical neural transmission and communication involves the release of neuronal action potentials, these action potentials and their accompanying electrical fields have been seen to be altered by drugs. EEG signals acquired from the scalp region have been seen to contain information regarding the state of the cortical and subcortical regions in the brain and can be used to decode levels of consciousness under anaesthetics, which typically involves the interpretation of the produced neural burst suppression patterns [14].

Despite the potential for EEG to serve as a means of tracking state of consciousness, there exist shortcomings which have constrained its widespread adoption in the area of anaesthesiology, and include the lack of standardised EEG indices for adults and children and inter‐patient variability, also as the EEG indices are generalised they assume that anaesthetic agents all have the same pharmacodynamic properties, whereas this is far from the case [15, 16]. Another source of interference is inter‐agent pharmacodynamic variability producing inaccuracies in EEG signal interpretation with existing models—particular examples include ketamine and nitrous oxide—plus the effect of adjuvant drugs [15, 16]. This warrants the need to be able to produce anaesthetic‐specific EEG models which can provide a customised decoding of a patient's neural state based on the kind of anaesthetic administered [15].

**FIGURE 2:**
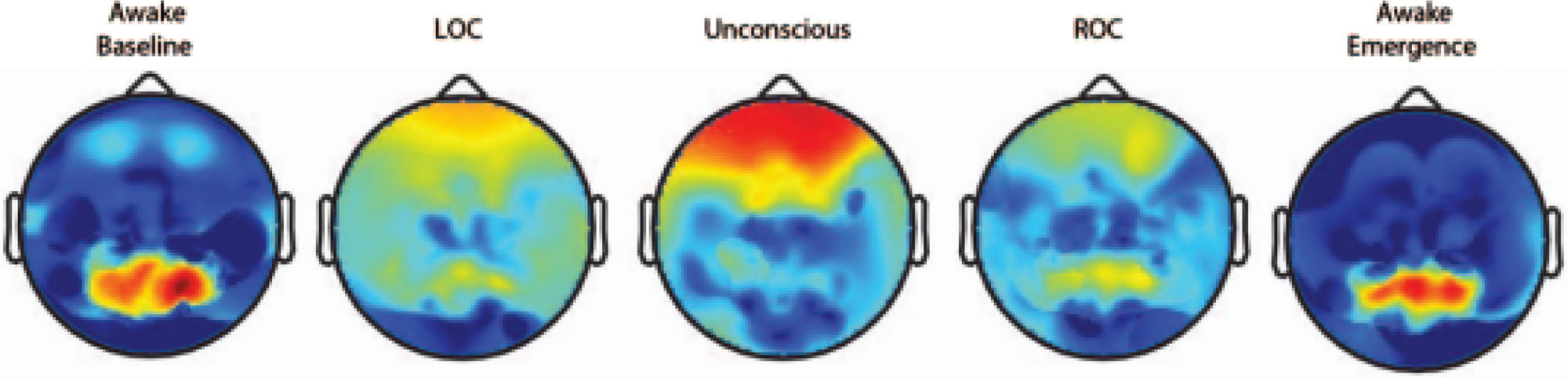
Neurological heatmaps showing the change of the bioelectrical field within the brain, from administration of propofol through to reawakening (where LOC is loss of consciousness and ROC is return of consciousness) [15]

A series of neurological heatmaps showing the change of the bioelectrical field within the brain, from the administration of propofol all the way towards the recovery of consciousness and eventual awakening, can be seen in Figure 2. During the awake stage there exist high neural oscillations around the occipital region which slowly shift and diffuse towards the frontal lobe and then go into a state of reverse back to the occipital region as soon as the patient is fully awake [15].

The bispectral index (BIS) is based on the EEG electrical field signal behaviour within the brain and is accepted as the standard means for anaesthetic depth monitoring, as produced by Aspect Medical Systems, Newton, MA, USA. The device has received Food and Drug Administration (FDA) approval and exhibits high accuracy in the monitoring of the GABA receptor anaesthetic [12, 16]. The scale for the BIS ranges from 0 (low brain activity) to 100 (fully awake state). The algorithm behind the BIS is not disclosed to the scholarly world due to proprietary rights; however, it has been seen that the BIS is limited in monitoring certain kinds of anaesthetics, it performs poorly on infants and in paediatrics, in addition to patients with neurological impairments and therein an altered neural circuitry due to neuroplasticity [12, 17]. To overcome this limitation, we aim to harness the capability of signal processing and intelligent learning methods towards the design of patient and anaesthetic agent‐specific models for the prediction.

The utilisation of machine learning models in medicine spans the use of models such as decision trees, discriminant analysis, support vector machines and artificial neural networks [18]. These models serve as a means towards decision support and are capable of high accuracy pattern recognition, upon being trained by the relevant training examples [18]. The machine learning models are fed a matrix of extracted features from what typically is a set of physiological time‐series data acquired from a patient. To ensure that the features are of a high quality and allow for the effective modelling of a physiological time‐series, signal decomposition methods are also frequently applied as a form of preprocessing prior to feature extraction. Signal decomposition methods allow for the deconvolution of a candidate signal into constituent parts, which help towards the reduction of uncertainty within the source signal. The approach has proved to be useful in areas spanning economics, seismic exploration and in the analysis of physiological signals, where the optimal region within the decomposed source signal can be said to be the region which contains the reach information relevant towards estimating/predicting the entity of interest [19, 20].

In this paper, a number of multiscale decomposition methods were applied and contrasted towards an effective anaesthetic depth prediction. This was done alongside a set of feature extraction exercises comprising a convolutional neural network and the deep wavelet scattering. The machine learning aspect involved the use of the linear discriminant analysis (LDA), which has been viewed as a computationally effective means towards pattern recognition, as seen in the bionic prosthesis literature [17].

Explicitly speaking, the contributions of this manuscript are as follows:

‐ A design of patient‐specific anaesthesia depth prediction, while also contrasting between multiscale signal decompositions and feature extraction methods for the discrete prediction of anaesthesia depth using the LDA classifier.
‐ An evaluation of the computation time of the designed methods alongside their various accuracies.

## 2 MATERIALS AND METHODS

### 2.1 Experimental data

The anaesthetic data used in this paper was taken from the opensource data which is freely available from Liu et al. [12], and was collected from patients who underwent minor surgery, that is, it did not involve for example the brain, heart, or lung etc [12]. The data was collected at the National Taiwan University Hospital (NTUH) where it also received ethical approval, and patients provided a written and informed consent prior to the data collection [12.]. Average statistics from the cohort who took part in the data collection process include: Age (years): 44.5 ± 12.9, Height (cm): 164.2 ± 7.1, Weight (kg): 63.4 ± 14.8, BMI: 23.4 ± 4.2, Operation timespan (min): 126.4 ± 72.9 (Supporting Information).

The patients were required to fast for 8 h prior to the surgery; and during the study, intravenous propofol was used for induction of anaesthesia alongside administration of the muscle relaxant, as appropriate [12]. As soon as patients lost consciousness (deemed by the lack of response to verbal commands), the next stage commenced, which comprised either propofol for swift short‐duration surgeries or inhalational anaesthesia, alongside air and oxygen for maintenance of anaesthesia, and were adjusted as appropriate by 1–1.5 minimal alveolar concentration [12]. Towards the end of the surgery, drugs such as Vagostin and morphine were also administered. A comprehensive list of the anaesthetic agents used for the various patients is documented in Table 1.

**TABLE 1:** List of anaesthetic agents used (further details regarding doses administered can be seen in Liu et al. [12])

Induction phase	Maintenance	Additional medication administered
Propofol	Sevoflurane	Morphine
Fentanyl	Desflurane	Ketamine
Atropine	Propofol	Atropine
Nimbex		Vagostin
Xylocaine		

**FIGURE 3:**
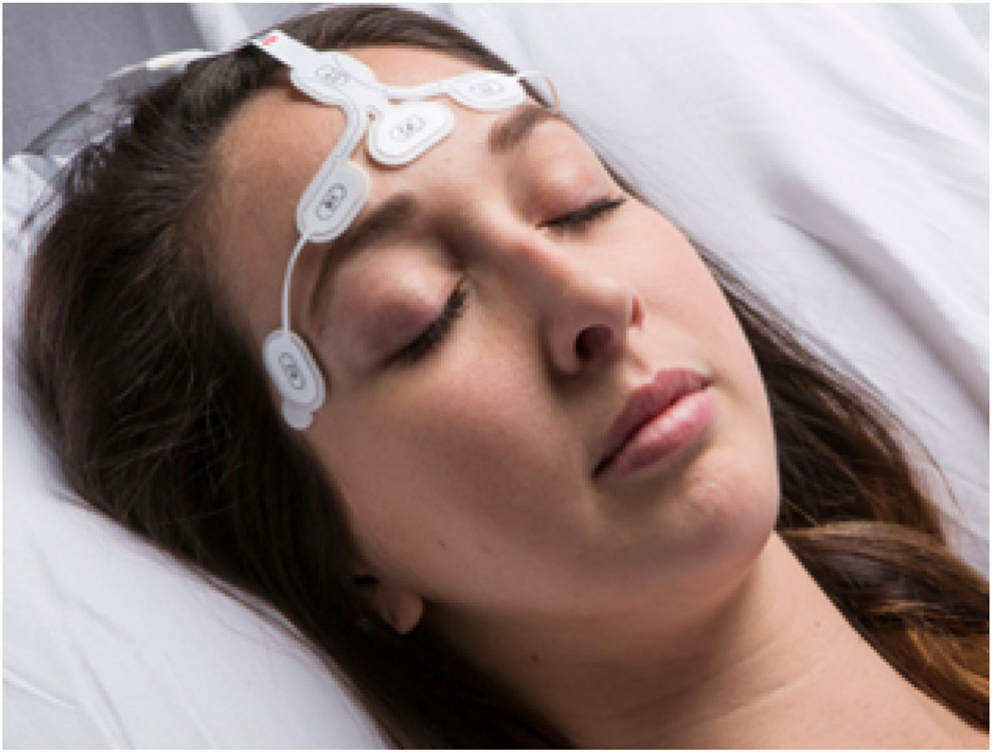
Sample EEG monitoring process during sedation [21]

In addition to the EEG monitors, standard physiological monitors such as electrocardiography, photoplethysmography, blood pressure, and pulse rate were measured during the process, where if any irregular fluctuations occurred, the doctors adjusted as appropriate. Figure 3 shows a sample EEG monitor during an anaesthetic sedation process, with the EEG electrodes across the forehead of the patient [12].

For the collection of the data used in this study, the EEG BISTM Quatro sensor with a single channel by Aspect Medical Systems, Newton, MA, USA, was used. The raw EEG waveforms, sampled at 128 Hz, were saved and acquired on a computer with Borland C++ Builder 6 software. The intermittent BIS, which serves at the ground truth label for consciousness in this work, was also used during the data acquisition, where values were computed and obtained every 5 s.

In this paper, four discrete consciousness states were investigated for prediction, namely; fully awake (80–100 BIS), semi awake (60–80 BIS), operative (40–60 BIS) and very low brain activity (0–40 BIS), which were determined using the BIS as the ground truth. For each consciousness class, 20,000 data samples were extracted from the time‐series of each of the 10 patients whose data were being used as part of the pilot work done in this paper, which were segmented into sub‐samples of 5000 to serve as repetitions for each class. This subset of patients’ data was used as part of the signal processing exercise to allow for a deep contrast of signal processing methods for each patient's EEG data, of which five different approaches were taken, as can be seen in Section 3.

The division of the data in this manner was done to ensure that each subsample repetition was robust enough and of a sufficient length to contain high integrity time‐series information to use for the subsequent signal processing exercises.

### 2.2 Signal processing and feature extraction

#### 2.2.1 Signal decomposition methods

- Noise‐assisted multivariate empirical mode decomposition (NA‐MEMD)

The empirical mode decomposition (EMD) is an established data‐driven signal decomposition approach for nonstationary time‐series signals, where the source signal is decomposed and expressed as a linear combination using a basis function, referred to as a set of intrinsic mode functions (IMFs) [22, 23]. Here, the IMF basis functions can be characterised as zero‐mean amplitude‐frequency modulated signals which ensure that the Hilbert transform/Hilbert‐Huang transform produces meaningful estimates of the input signals [22, 23]. Due to necessity, algorithmic extensions have been made to the EMD algorithm to facilitate multivariate processing and are referred to as multivariate EMD (MEMD) [22, 23]. The decomposition behaviour of the EMD has been closely likened to that of the wavelet decomposition with their respective filter bank similarities, while in the multivariate case the filter bank structure takes an overlapping frequency format due to the presence of multiple channels, in order to link separate components of a multivariate signal [22, 23]. Based on the ability to align sub‐bands of various frequencies amidst noise with the MEMD method, the noise‐assisted MEMD (NA‐MEMD) has been devised by ur Rehman and Mandic, which utilises an almost pseudo‐dyadic set of filter banks in the decomposition process, and ultimately is capable of aligning the oscillatory modes in the IMF from various channels, which has been seen to minimise the ‘mode mixing problem’ from channel components in the multivariate IMF format [22, 23]. A summary of the algorithmic steps involved in the implementation of the mentioned variants of the EMD can be seen in ur Rehman and Mandic [22].

The various parameters used for the implementation of the NA‐EMD include the number of channels/windows as 4, projection directions as 8 (i.e., number of windows × 2), the stop vector tolerances as [0.075 0.75 0.075], and the noise intensity as 1.

- Linear series decomposition learner (LSDL)

The LSDL represents a metaheuristic signal decomposition method devised by Nsugbe et al., originally for source separation exercises comprising micron scale particle mixtures and high frequency acoustic signals [20, 24–26]. The decomposition method is computationally efficient and works in the time domain with a set of heuristically tuned linear thresholds as a basis function, in addition to a peak identification sequence—which together ultimately yields a series of decomposed signals based on amplitude bands—followed by a learning process which is performed via a performance index that is used to select an optimal region from the candidate decompositions [20, 24–26]. The decomposition method has seen subsequent application in various aspects of clinical medicine involving the analysis of nonlinear and stochastic physiological signals such as rehabilitation, pregnancy medicine, and more recently in the prediction of adolescent schizophrenia from EEG signals [20, 27, 28].

**FIGURE 4:**
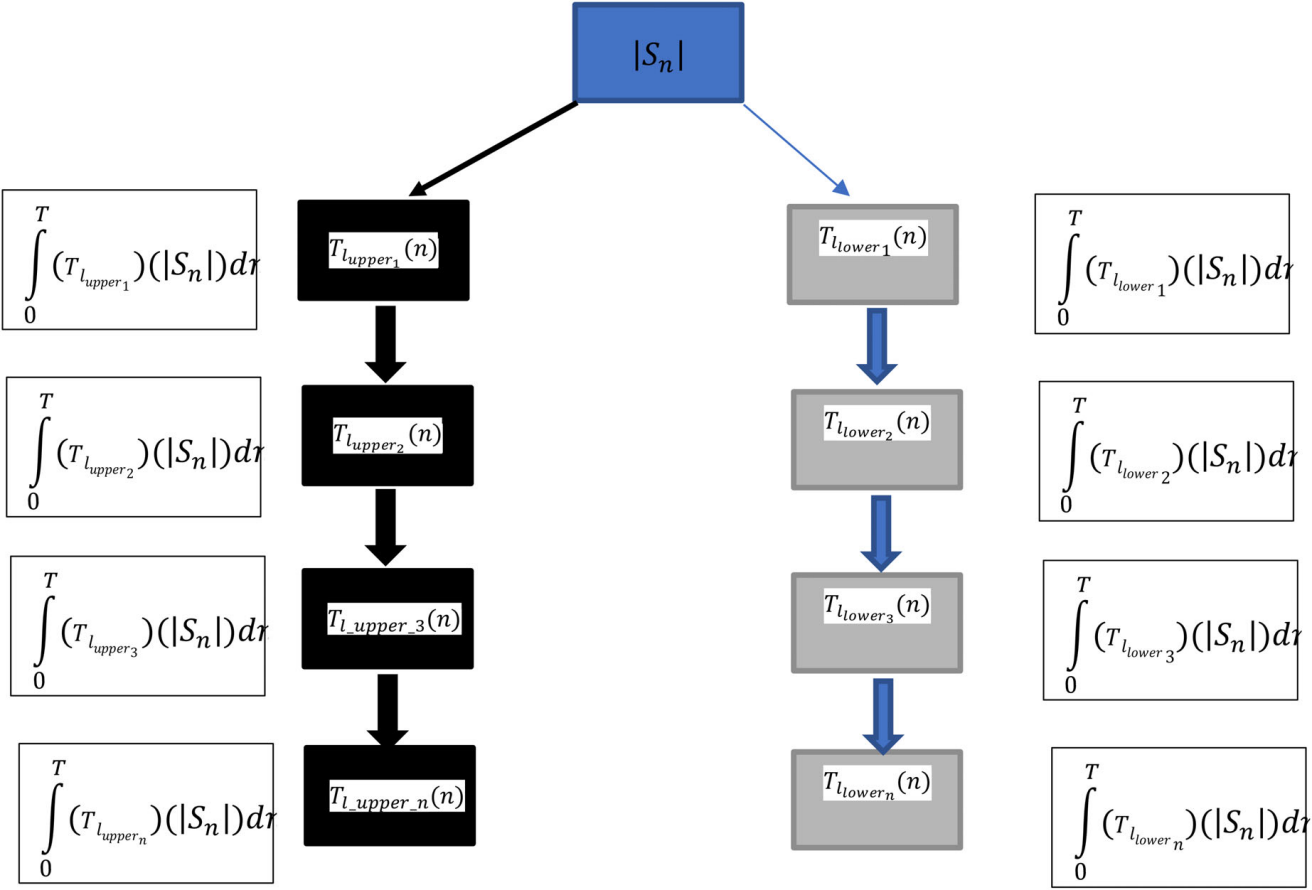
Decomposition tree representation for the LSDL (where *T* indicates the length of the candidate signal) [28]

The full list of heuristics used in performing the LSDL decomposition can be seen in Nsugbe et al. [20] and [26]. The parameters used in the tuning of the LSDL thresholds for the work can be seen in Table 2 for an absolute signal $|S_{n}|$, while a tree‐like flow of the decomposition process can be seen in Figure 4.

**TABLE 2:** Threshold parameters for the LSDL

Iteration	1	2	3	n
Upper threshold region parameter (Upper)	$T_{l\_upper\_1}=50\%\;\mathrm{of}\;\max\left|S_{n}\right|$	$T_{l\_upper\_2}=\frac{\max|S_{n}|+T_{l\_upper\_1}\;}{2}$	$T_{l\_upper\_3}=\frac{\max|S_{n}|+T_{l\_upper\_2}\;}{2}$	$T_{l\_upper\_n}=\frac{\max|S_{n}|+T_{l\_upper\_n-1}\;}{2}$
Lower threshold region parameter (Lower)	$T_{l\_lower\_1}=50\%\;\mathrm{of}\;\max\left|S_{n}\right|$	$T_{l\_lower\_2}=\frac{T_{l\_lower\_1}}{2}$	$T_{l\_lower\_3}=\frac{T_{l\_lower\_2}}{2}$	$T_{l\_lower\_n}=\frac{T_{l\_lower\_n-1}}{2}$

Where $T_{l\_upper\_n}$ and $T_{l\_lower\_n}$ are the thresholds corresponding to the upper and lower amplitude regions of the signal, respectively.

Mathematically speaking, the decomposition series can be expressed as follows:

(1) $$\begin{array}{ccc} \left|S_{n}\right| & = & \left(\int_{0}^{T}(T_{l_{upper_{1}}}\right)\left(\left|S_{n}\right|\right)dn+\;\int_{0}^{T}\left(T_{l_{upper_{2}}}\right)\left(\left|S_{n}\right|\right)\\ & & \text{…}\;..\int_{0}^{T}\left(T_{l_{upper_{n}}}\right)\left(\left|S_{n}\right|\right)dn)+\left(\;\int_{0}^{T}({T_{l_{lower}}}_{1}\right)\left(\left|S_{n}\right|\right)dn\\ & & +\;\int_{0}^{T}\left({T_{l_{lower}}}_{2}\right)\left(\left|S_{n}\right|\right)dn\text{…}..\int_{0}^{T}\left({T_{l_{lower}}}_{n}\right)\left(\left|S_{n}\right|\right)dn\;) \end{array}$$

(2) $$\left|S_{n}\right|=\int_{0}^{T}(Upr_{n}\left(\left|S_{n}\right|\right)+\int_{0}^{T}(Lwr_{n}\left(\left|S_{n}\right|\right)$$

- Deep wavelet scattering (DWS)

The DWS is an approach that decomposes and subsequently follows this with a multiscale unsupervised feature extraction; the method works quite well with a constrained set of samples and does not need to learn filter parameters from the data, which contributes to its effectiveness with a constrained set of samples [29, 30]. The properties exhibited by the DWS—which encompass that of the wavelet transform and the convolutional neural network (CNN)—that allow for effective feature extraction, are chiefly as follows: multiscale contractions, the linearisation of hierarchical symmetries, and sparse representations [29, 30].

For a given signal f(t),analysed by a filter Ø, and a wavelet Ψ, ${Ø}_{J}\left(t\right)$ is a localised translation invariant low pass filter with a scale defined as *T*, for a range of frequencies. The DWS consists of a frequency resolution $Q_{k}$ denoted as $\wedge_{k}$ with a multiscale high pass filter bank ${\left\{\Psi_{jk}\right\}}_{jk\in \wedge_{k}}$ assembled through the dilation of a wavelet Ψ. The convolutions that take place can be defined as $S_{0}f\left(t\right)=f*{Ø}_{J}\left(t\right)$, where *S*_0_ is the (initial) zero‐order scattering coefficient that creates a local translation invariant set of features of *f* that can be recovered through a modulus transform $|W_{1}|$, as in $|W_{1}|f={\left\{S_{0}f\left(t\right),\left|f*\Psi_{j1}\left(t\right)\right|\right\}}_{j1\in \wedge_{1}}$. In an iterative fashion, the first order scattering coefficients are obtained through the averaging of the wavelet modulus coefficient with ${Ø}_{J}\left(t\right)$, as follows:

(3) $$S_{1}f\left(t\right)={\left\{\left|f*\Psi_{j1}\left(t\right)\right|*{Ø}_{J}\left(t\right)\right\}}_{j1\in \wedge_{1}}$$

The lost information from the averaging process is recoverable via the use of the wavelet modulus $|W_{2}\left|\right|f*\Psi_{j1}|={\left\{S_{1}f\left(t\right),||f*\Psi_{j1}|*\Psi_{j2}\left(t\right)|\right\}}_{j2\in \wedge_{2}}$, from which the second order coefficients are subsequently defined as $S_{2}f\left(t\right)={\left\{||f*\Psi_{j1}|*\Psi_{j2}|*{Ø}_{J}\left(t\right)\right\}}_{j1\in \wedge_{1}}i=1,2.$

**FIGURE 5:**
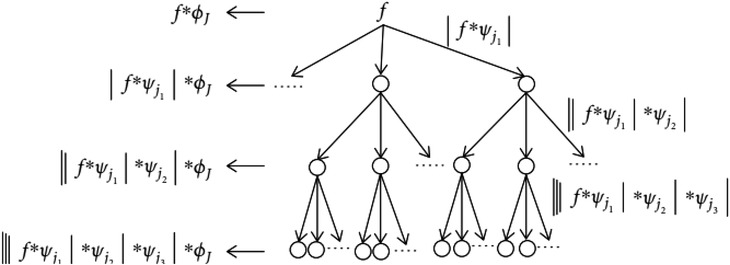
Wavelet scattering decomposition tree [31]

The outcome of the WSD is a scatter matrix $Sf\left(t\right)={\left\{S_{m}f\left(t\right)\right\}}_{0\leq m\leq l}$, which concatenates all obtained coefficients as a means towards characterising the signal with a decomposition depth *l*. A tree‐like projection of the WSD can be seen in Figure 5.

The bulk of the energy of the scatter coefficients has been said to reside in the first two layers of the decomposition, thus only two layers were considered in this work. Alongside this, the other parameters used as part of the WSD include the Gabor wavelet, an invariance scale of 1 s, and the wavelet filter banks of 8 wavelets per octave for the first filter bank, succeeded by 1 wavelet per octave in the second layer.

#### 2.2.2 Feature extraction methods

Two feature extraction methods were used in this study, one involving a select list of handcrafted features, while the other involved an automated means via a candidate deep learning architecture.

- Handcrafted features

These comprised a concatenation of linear, frequency and non‐linear features, which have shown capability to model stochastic physiological signals, as per prior studies [17, 32]. The list of features is as follows:

Linear: Mean absolute value, waveform length, zero crossing, root mean square, 4th order autoregressive coefficient, number of signal peaks, simple squared integral, and variance. The threshold value of 1 μv was selected for all features requiring thresholds and in the case of the signal peaks.

Frequency: Maximum cepstrum coefficient, and median frequency.

Non‐linear: Sample entropy, maximum fractal length, Higuchi fractal dimension, and detrended fluctuation analysis. The parameters used in the calculation of the non‐linear features were 2 and 0.2 for the values of *m* and *r* for the sample entropy, and *k* as 10 for the Higuchi fractal dimension.

- Deep‐learning (DL) features

**FIGURE 6:**
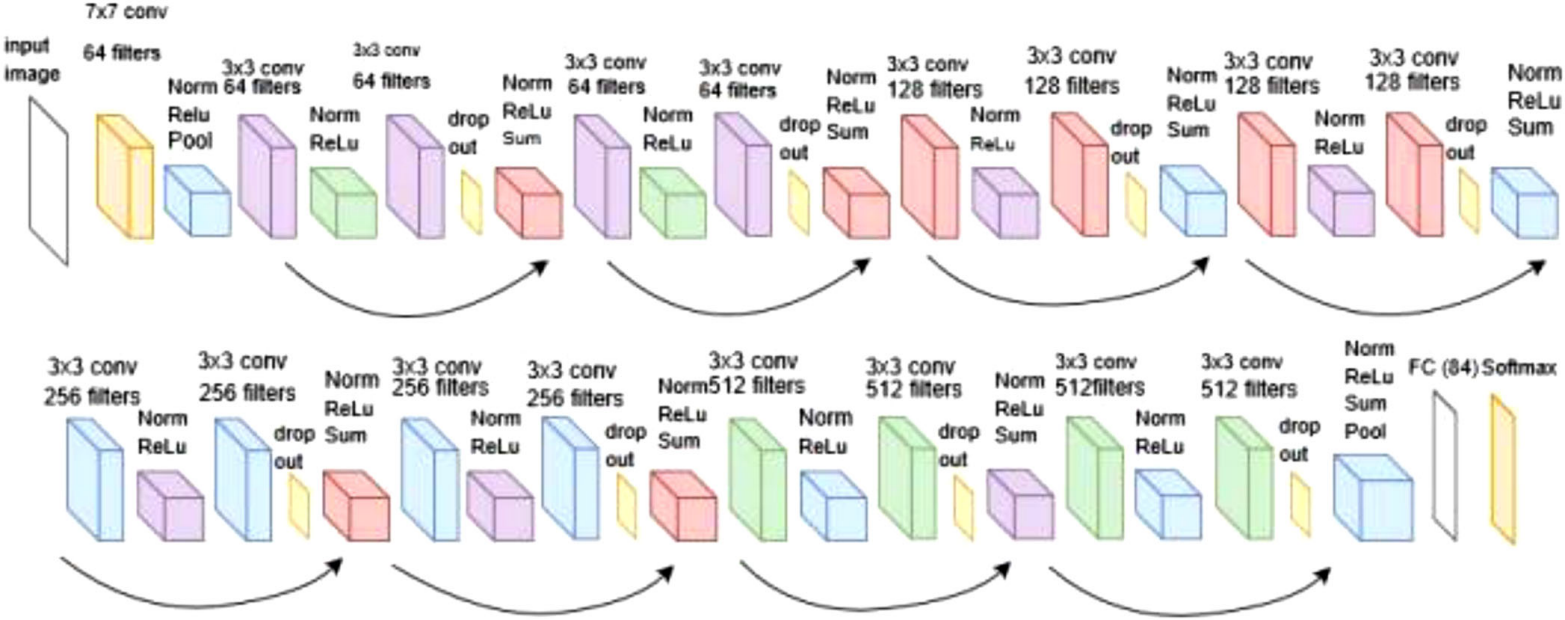
Schematic of the ResNet18 architecture [34]

The ResNet18 DL architecture was chosen in this case, as motivated from previous work due to its relative computational effectiveness and lower dimensional features [33]. This architecture is inspired from the pyramidal cells in the human brain where connections are skipped, and from a computational perspective this helps to minimise any network overfit, in addition to the vanishing gradient problem [33]. An image of the ResNet18 architecture can be seen in Figure 6.

### 2.3 Machine learning method

- LDA

The LDA is a computationally effective machine learning classification method based around the projection of high dimensional data into a lower subspace where class boundaries are implemented [17]. The linear variant of the discriminant analysis was used in this work, and the discriminant function can be expressed as Equation (4)

(4) $$D_{l}\left(x\right)=\mu_{c}^{T}\sum_{l}^{-1}x-1/2\mu_{c}^{T}\sum_{l}^{-1}\mu_{c}$$

where ð*_l_* symbolises the discriminant function, $\mu_{c}^{T}$is the mean vector for a specific data class *c*, *x* is a sample from within a feature vector, and $\Sigma_{l}$ is the pooled covariance matrix. The validation process of the classifier involved a 1 × *k*‐fold cross‐validation method where *k* was chosen as 10.

## 3 RESULTS AND DISCUSSION

### 3.1 NA‐EMD

The IMFs 2 and 3 were deemed as the optimal modes from the NA‐EMD, as indicated in previous work, which was taken on the assumption that these modes are optimal and generalisable across all patient subjects [12]. The results for the NA‐EMD alongside the Handcrafted Features can be seen in Table 3, where a broad range of results can be observed depending on the patient. The variations in results provides evidence of the distinct neural circuitry which exists between patients, hence why patient specific models are a proposed better fit in this area of study. Overall, the NA‐EMD appears to be able to predict the DoA in a varied sense, with the results ranging from 62.5–87.5%, potentially implying that a signal decomposition approach may not be ideal in this type of study due to the need to infer depth of sedation and consciousness from varied frequency regions, as supposed to a fixed band.

**TABLE 3:** Result of the NA‐MEMD and handcrafted features

Subject number*	Classification accuracy (%)
1	62.5
2	71.9
3	84.4
4	71.9
5	87.5
6	71.9
8	71.9
10	65.6
11	68.8
12	78.1
Mean classification accuracy	73.45 ± 7.83

^*^Where the subject numbers reflect the order of the datafiles used from the opensource EEG data files published in Liu et al. [12].

### 3.2 Raw signal

This case involved the use of the full signal without any form of decomposition preprocessing of the signal prior to the extraction of handcrafted features. The results, using a combination of the raw signal and handcrafted features, can be seen in Table 4, and appear to be notably improved in comparison with the NA‐MEMD. The use of the raw signal helps to demonstrate the broadband frequency nature of the EEG signal induced by the varied levels and degrees of consciousness during the surgery, which thus results in the quality of information in the signal being spread throughout it, as opposed to being primarily focused within a fixed frequency band. In line with the results from the NA‐MEMD, a broad and varied range of classification results were obtained from the raw signal, but with an improved classification accuracy.

**TABLE 4:** Results of the raw signal and handcrafted features

Subject number	Classification accuracy (%)
1	81.3
2	68.8
3	87.5
4	100
5	93.8
6	93.8
8	93.8
10	75.0
11	75.0
12	87.5
Mean classification accuracy	85.65 ± 10.23

### 3.3 LSDL

In this implementation of the LSDL, two decomposition regions were used for the signal processing exercise (like the NA‐MEMD) due to the perceived bad frequency band behaviour of the EEG signal during variations in consciousness. The results of the LSDL can be seen in Table 5, where the optimal decomposition regions (highlighted in bold) were selected for both the Upper and Lower decomposition regions.

**TABLE 5:** LSDL decomposition results

	Iteration 1	Iteration 2	Iteration 3	Iteration 4
Upper	2.825	2.824	2.682	2.806
Lower	2.828	2.828	2.828	2.821

The results from the signal processing exercise involving the aforementioned optimal decomposition regions from the LSDL, alongside the extraction of handcrafted features, can be seen in Table 6. Here the LSDL was able to produce a high classification accuracy of the DoA, which is on par with the raw signal and notably higher than the NA‐MEMD.

**TABLE 6:** Results of the LSDL and handcrafted features

Subject number	Classification accuracy (%)
1	81.3
2	81.3
3	81.3
4	100
5	75.0
6	81.3
8	93.8
10	68.8
11	81.3
12	100
Mean classification accuracy	84.41 ± 10.3

Although a broad variation of the results is still apparent, it can be said that the LSDL is better suited to decomposing signals of this kind when benchmarked against the NA‐MEMD. In addition to a high classification accuracy, the LSDL can also be assumed to be computationally effective due to the requirement for a reduced number of samples post‐decomposition and prior to making a prediction, when compared to the raw signal, which uses a full windowed sample. Further insights into the computational performances of the various methods are discussed subsequently in Section 3.6 (Computation time results). However, a pre‐learning and calibration phase is required to determine the optimal decomposition regions.

### 3.4 DWS

As described, the DWS is a method that is capable of both decomposing a candidate signal alongside an extraction of deep features, via means that are all unsupervised. The results for the DWS can be seen in Table 7, and are slightly behind those of the raw signal and LSDL, although the standard deviation is much less, insinuating a more stable performance across subjects and less sensitivity to the EEG signals from the varied neural circuitry of the various subjects. Although the work is firmly based around the development of a unique anaesthesia model for each patient, the minimal standard deviation experienced per patient makes the DWS a good candidate for a generalised DoA prediction framework, similar to the way the BIS is designed. However, the major downside of the DWS remains the fact that its configuration is largely unsupervised, thus its features lack interpretability when contrasted with handcrafted features.

**TABLE 7:** Results of the DWS

Subject number	Classification accuracy (%)
1	75.8
2	84.1
3	79.0
4	83.1
5	75.6
6	76.8
8	92.5
10	71.6
11	78.0
12	84.1
Mean classification accuracy	80.06 ± 5.98

### 3.5 DL

The results from the ResNet18 unsupervised feature extraction and classification can be seen in Table 8. These results were obtained using the deep features in the network, where it can be seen that the results are the lowest amongst all the classification exercises conducted. This is thought to be due to the network architecture which offers a small size of features. This could be expanded towards a larger DL architecture with more features, but would come at the price of further computation complexity. Thus, the candidate DL method would not be an appropriate choice for the case study being considered in this paper.

**TABLE 8:** Results of the DL

Subject number	Classification accuracy (%)
1	68.8
2	81.3
3	68.8
4	75.0
5	81.3
6	62.5
8	n/a
10	62.5
11	81.3
12	75.0
Mean classification accuracy	72.94 ± 7.67

The model performance in Table 9 shows a ranking of the total amount of times each model produced the highest classification accuracy amongst the 10 patient subjects. The results involving the raw signal provided the best classification accuracy 7 times, followed by the LSDL with 5, the DWS and DL with 1 each, and 0 for the NA‐MEMD, therein showing a case of model optimality between the raw signal and LSDL, as previously mentioned.

**TABLE 9:** Method performance analysis (bold indicates a joint performance matching another method)

	NA‐EMD	Raw signal	LSDL	DWS	DL
Subject 1		X	X		
Subject 2				X	
Subject 3		X			
Subject 4		X	X		
Subject 5		X			
Subject 6		X			
Subject 8		X	X		
Subject 10		X			
Subject 11			X		X
Subject 12			X		
Total	0	7	5	1	1

Table 10 provides a summary result and perspective on the various methods, where once again the raw signal and LSDL produced the best classification accuracies, albeit with a relatively high standard deviation value. The DWS produced the smallest standard deviation amongst the various methods, with a classification accuracy which is closely comparable to that of the methods involving the Raw Signal and the LSDL, thereby making it a good candidate for a potential generalised DoA prediction method, if necessary.

**TABLE 10:** Method contrast table

Method	Classification accuracy (%)
NA‐MEMD + Handcrafted features	73.45 ± 7.83
Raw Signal + Handcrafted features	85.65 ± 10.23
LSDL + Handcrafted features	84.41 ± 10.30
DWS	80.06 ± 5.98
DL	72.94 ± 7.67

It can be said that the certain cases where a low classification accuracy was obtained with each method could be attributed to the infeasibility of the method to predict DoA amidst the presence of the contents of the anaesthetic formulation dosed to the patient, which was seen to vary slightly between patients. It has not been possible to investigate this further due to the accompanying information provided with the dataset.

### 3.6 Computation time results

Five key computational metrics of the various methods, aside from the classification accuracy, were computed and can be seen in Table 11. The LSDL appears to have a much quicker decomposition timeframe for decomposing a sample signal, in line with its optimal architecture established in previous studies. The average number of samples prior to feature extraction is largely consistent for all methods except for the LSDL and NA‐MEMD (due to the prior decomposition actions), where it can be seen that the LSDL uses the lowest number of samples to perform its subsequent computations, which may carry benefits in terms of data storage infrastructure. The feature extraction times show that the DWS and CNN benefit from their unsupervised feature extraction and possess the quickest computation times, while there is a similar feature extraction computation time for the raw signal and the LSDL, with the NA‐MEMD having the longest feature extraction computation time. The classifier prediction time appears to be largely consistent for all five methods due to the choice of a low complexity classifier. Despite their longer computation times, the raw signal and LSDL methods continue to be the optimal methods when combined with their strong classification performances for use in DoA, based on the results. The bulk of the computation time for all methods appears to be embedded within the feature extraction stage; thus, to further streamline the computation time, optimisation exercises can be conducted to downselect for the most optimal set of features while retaining maximal classification accuracy.

**TABLE 11:** Computational metrics results

	Signal decomposition (s)	Average number of samples (prior to feature extraction) (s)	Feature extraction (s)	Classifier prediction time (LDA)*Using a sample from a patient's EEG signal (s)	Total (s)
NA‐MEMD + Handcrafted features	12.59 ± 0.26	40,000 (IMF 2 and 3)	80.4 ± 9.60	0.01 ± 0	90.3 ± 9.86
Raw signal + Handcrafted features	n/a	20,000	9.60 ± 0.28	0.01 ± 0	9.61 ± 0.28
LSDL + Handcrafted features	0.04 ± 0.02	1150 (for two optimal thresholds)	12.80 ± 0.48	0.02 ± 0	12.86 ± 0.5
DWS (Unsupervised feature extraction)	n/a	20,000	5.20 ± 2.00	0.01 ± 0	5.21 ± 2.0
CNN (Unsupervised feature extraction)	n/a	20,000	2.90 ± 0.25	0.12 ± 0.04	3.02 ± 0.29

*Computation times were conducted based on a single patient's dataset, where the metrics were computed with a laptop of Intel(R) Core(TM) i5‐3210 M CPU @ 2.50G Hz, with a 64‐bit operating system and a 6GB RAM.

## 4 CONCLUSION

Anaesthetics are neurotropic substances that are used in surgical processes to induce a regulated state of consciousness loss. A theory around their workings is based around the nulling of the feedback pathway of the brain (as shown in Figure 1), which is also backed by research showing feedback connectivity attenuation in various portions of the brain. Despite the longstanding use of global anaesthetics for surgical purposes, there continues to be reported side effects around their use, including lingering cognitive dysfunction, which makes it paramount for the effective regulation of the DoA administered during surgical endeavours.

The BIS is a common means of monitoring anaesthetic DoA, but its ability to closely track cognitive state is hindered when certain anaesthetic agents are used. Thus, the work done in this paper is a pilot study aimed to overcome this using EEG neural oscillation signals recorded during anaesthesia from 10 patient subjects, alongside signal processing and machine learning methods. In particular, this work explored the design of patient‐specific models to predict discrete DoA states using various methods while using the BIS readings as a ground truth label.

The results showed that the raw signal and the LSDL were seen to be the most optimal methods, as determined via the classification accuracies obtained, while their computation times were also seen to be reasonable for a possible real‐time performance capability. With a potential view towards a generalised DoA prediction method such as BIS, the DWS is proposed to be the best fit due to its concise standard deviation prediction band alongside low computational complexity metrics. Given the nature of the proposed patient‐specific approach, it is anticipated that a real‐time model ‘System Identification’ process is to be conducted at the start alongside an anaesthetist, where the computational model builds a patient‐specific model, like the proposed approach done by Nsugbe et al. [35].

Potential areas of subsequent work include the validation of the best performing signal processing methods on a larger cohort of patients’ data, the application of nonlinear classification methods, the extension of this approach to a continuous means of estimating DoA using regressions, model validation on paediatrics, elderly, epileptics etc., and also including subjects from various demographics.

## CONFLICT OF INTEREST

The authors declare no conflict of interest.

## Supporting information



Supporting Information

## Data Availability

The data is available from a cited repository within the manuscript.
