# Supplementary material for: Multiscale depth of anaesthesia prediction for surgery using frontal cortex electroencephalography
Source: Healthc Technol Lett. 2022 May 3;9(3):43–53. doi: 10.1049/htl2.12025 (PMC9160818; doi:10.1049/htl2.12025)
Supplement: Supplementary file 1 — Supporting Information [file HTL2-9-43-s001.docx]

**5. Supplementary Material**

**5.1. Theoretical Model of the Effects of Anaesthesia on the EEG**

Although this work is not based on theoretical simulations, here a neural field model is articulated and described by Liley et al. to mathematically describe the onset of resting state EEG waves in a generalised manner, whose purpose is to quantitatively serve as an alternative means for better appreciation of the onset of heterogenous neural burst suppression manifested within the cortex [36,37]. The assumptions made in Liley et al.’s model include a 2-dimensional toroidal geometry, isotropic and homogenous connectivity alongside partial differential equations (PDEs) [36,37]. Liley et al.’s model considers the use of the damped wave PDEs, which are known to be a prime mechanism for the spread of events within neural field models [36,37].

Liley et al.’s model is used as the theoretical basis in this section and is assembled at the scale of the cortical microcolumn, where within each hypothetical column there exist various excitatory and inhibitory neurons interacting with each other in a feedforward and feedback nature, and where the macrocolumns themselves interact with each other via long range axonal fibres, as can be seen in Figure 7 [36,37].


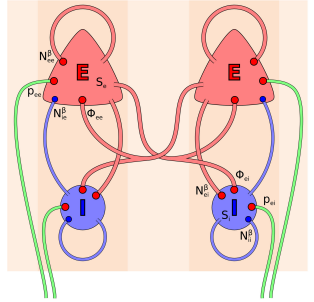


**Figure 7:** A connective network view of the proposed model by Liley et al., comprised of Excitation-E and Inhibitory-I neural populations, where each subpopulation can be assumed to be a microcolumn [36,37]. External cortical inputs have been depicted by green fibres. The supporting symbols expressed as part of the diagram can be seen in equation 5.

As part of this model, the cortical activity is denoted via the spatial temporal evolution of the mean excitation field and inhibitory soma membrane potentials, mathematically represented as $h_{e}(\overset{⃑}{x},t)$ and $h_{i}(\overset{⃑}{x},t)$. The link between this model and the EEG signal, which represents electrophysiological activity, is via $h_{e}$, which has been said to be correlated to EEG signals [38]. The presence of excitatory and inhibitory neurons is mathematically modelled in the form of spatially averaged conducting neurons, as follows:

$\frac{\tau_{k}\partial h_{k}(\overset{⃑}{x},t)}{\partial t}=h_{k}^{r}-h_{k}\left( \overset{⃑}{x},t \right)+\sum_{l=e,i} \frac{h_{lk}^{eq}-h_{k}\left( \overset{⃑}{x},t \right)}{\left| h_{lk}^{eq}-h_{k}^{r} \right|}I_{lk}(\overset{⃑}{x},t)$ (5)

Here, $\overset{⃑}{x}\in\boldsymbol{R}^{2}$ is a spatial location along the cortical sheet, the subscripts $l,k$ $\in$ {*e*, *i*} are indicative of excitatory and inhibitory populations respectively, and the double subscripts represent the presynaptic and postsynaptic target. The parameters $h_{k}^{r}$ represent the mean resting membrane potential which decays exponentially with a characteristic timescale $\tau_{k}$, without any input source $I_{lk}$. In equation 5, the preceding fraction of $I_{lk}$ serves as its weighting factor to ensure that the depolarisation effects of any further excitation decays in a linear fashion. The weight of the resting potential can either be +1 for excitatory or -1 for an inhibitory input.

The oscillatory behaviour of the postsynaptic potentials (PSPs) $I_{lk}$ is represented in this case using a critically damped oscillator controlled by the mean rate of the excitatory and inhibitory signals from the axons $A_{lk}$, and defined as follows, as per Liley et al.’s work [37].

$\left( \frac{1}{\gamma_{lk}}\frac{\partial}{\partial t}+1 \right)^{2}I_{lk}\left( \overset{⃑}{x},t \right)=\frac{eҐ_{lk}}{\gamma_{lk}}A_{lk}\left( \overset{⃑}{x},t \right)$ (6)

$A_{ek}\left( \overset{⃑}{x},t \right)=N_{ek}^{\beta}S_{e}\left[ h_{e}\left( \overset{⃑}{x},t \right) \right]+N_{ek}^{\alpha}\emptyset_{ek}\left( \overset{⃑}{x},t \right)+p_{ek}\left( \overset{⃑}{x},t \right)$ (7)

$A_{ik}\left( \overset{⃑}{x},t \right)=N_{ik}^{\beta}S_{i}[h_{i}\left( \overset{⃑}{x},t \right)]$ (8)

The three main sources of conductances for postsynaptic activities are local-($S_{e})$, cortico-cortical-($\emptyset_{ek})$, and subcortical-$(p_{ek})$; while in the case of the inhibitory conductances, the primary source is local-($S_{i})$, largely due to the thalmic and cortical inhibitory axons which are mostly short-range, as made apparent by neuroanatomical evidence [36,37]. At a point location, a presynaptics spike activity $A_{lk}=\delta(t)$ would ultimately yield an alpha response of the following form:

$I_{lk}\left( t \right)=\frac{eҐ_{lk}}{\gamma_{lk}}\alpha_{lk}\left( t \right)$ (9)

$\alpha_{lk}\left( t \right)=\gamma_{lk}^{2}te^{-\gamma_{lk}^{t}}\theta(t)$ (10)

Where $\theta(t)$ is the heaviside step function, $\alpha_{lk}$ is the alpha function, $\gamma_{lk}$ is the exponential decay of the PSP, $Ґ_{lk}$ is the maximum amplitude of the PSP, and $\gamma_{lk}$ is the rise time to peak amplitude. It can also be said that the conduction which occurs through a passive dentrite leads to a somewhat attenuated PSP with a notably lower magnitude. The variables $S_{e}$ and $S_{i}$ represents local mean excitatory and inhibitory firing, which are expressed to be sigmoidal functions of $h_{k}$, as

$S_{k}[h_{k}\left( \overset{⃑}{x},t \right)=S$^max^_k_$/\{1+exp[-\surd2\frac{h_{k}\left( \overset{⃑}{x},t \right)-\mu_{k}}{\sigma_{k}}]$}. The $S$^max^_k_ represents the maximum possible firing rate while $\mu_{k}$ and $\sigma_{k}$ represent the mean and standard deviation of firing thresholds of the distribution, which can be assumed to be normally distributed. The dynamical propagation of the pulses from the axon, denoted by $\emptyset_{ek}$, can crucially be modelled as a damped wave equation, thus:

$\left[ \left( \frac{1}{v_{ek}}\frac{\partial}{\partial t}+\frac{1}{\lambda_{ek}} \right)^{2}-\nabla^{2} \right]\emptyset_{ek}\left( \overset{⃑}{x},t \right)=\frac{1}{\lambda_{ek}^{2}}S_{e}\left[ h_{e}\left( \overset{⃑}{x},t \right) \right]$ (11)

Note that $\lambda_{ek}$ would comprise an inhomogeneous wave propagated with conduction velocity $v_{ek}$.

For an anaesthetic-specific mathematical description, readers are advised to consult Bojak et al. [36] and Liley et al. [37] for further details, where specifically a model is described around the isoflurane anaesthetic agent.

**References**

36. Bojak I, Stoyanov ZV, Liley DTJ. Emergence of spatially heterogeneous burst suppression in a neural field model of electrocortical activity. Front Syst Neurosci. 2015;9:18.

37. Liley DTJ, Cadusch PJ, Dafilis MP. A spatially continuous mean field theory of electrocortical activity. Network. 2002 Feb;13(1):67–113.

38. Bojak I, Breakspear M. Neuroimaging, Neural Population Models for. In: Jaeger D, Jung R, editors. Encyclopedia of Computational Neuroscience [Internet]. New York, NY: Springer New York; 2014 [cited 2022 Feb 27]. p. 1–29. Available from: http://link.springer.com/10.1007/978-1-4614-7320-6_70-1
